# Novel plasma microRNA expression features in diagnostic use for Epstein-Barr virus-associated febrile diseases

**DOI:** 10.1016/j.heliyon.2024.e26810

**Published:** 2024-02-23

**Authors:** YiFei Xu, Ying Chen, Qingluan Yang, Yuxiang Lu, Rui Zhou, Haohua Liu, Yanjie Tu, Lingyun Shao

**Affiliations:** a School of Traditional Chinese Medicine, Shanghai University of Traditional Chinese Medicine, Shanghai, 201203, People‘s Republic of China; b Department of Febrile Disease, School of Traditional Chinese Medicine, Shanghai University of Traditional Chinese Medicine, Shanghai, 201203, People‘s Republic of China; c Department of Infectious Diseases, National Medical Center for InfectiousDiseases, Shanghai Key Laboratory of Infectious Diseases and Biosafety EmergencyResponse, Huashan Hospital, Shanghai Medical College, Fudan University, Shanghai, 200040, People's Republic of China

**Keywords:** EBV, miRNA, EBV-HLH, Molecular diagnostics, Immune response

## Abstract

**Background:**

Epstein-Barr virus (EBV) is widely infected in humans and causes various diseases. Among them, microRNAs of EBV play a key role in the progression of EBV-associated febrile diseases. There're few specific indicators for rapid differential diagnosis of various febrile diseases associated with EBV, and the lack of more reliable screening methods with high diagnostic utility has led to spaces for improvement in the accurate diagnosis and efficient treatment of relevant patients, making EBV infection a complicated clinical problem. With recent advances in plasma microRNA testing, the apparent presence of EBV microRNAs in plasma can help screen for EBV infection. The gene networks targeted by these microRNAs can also indicate potential biomarkers of EBV-associated febrile diseases. This study aimed to identify some novel miRNAs as potential biomarkers for early diagnosis of respectively EBV-associated febrile diseases.

**Materials and methods:**

A total of 110 participants were recruited for this task. First, we performed high-throughput sequencing and preliminary PCR validation of differentially expressed miRNAs in 15 participants with EBV-associated fever (divided into common EBV carriers), infectious mononucleosis (IM) and chronic active EBV infection (CAEBV), EBV-associated Hemophagocytic Lymphohistiocytosis group (EBV-HLH), and 3 healthy individuals. After a comprehensive analysis, 10 miRNAs with abnormal expression were screened, and then qRT-PCR was performed in the rest of 95 participants to detect the validation of miRNAs expression in plasma samples. Thereafter, we further investigated their potential for clinical application in EBV-related febrile diseases by using a combination of Gene Ontology analysis, Kyoto Encyclopedia of Genes and Genomes pathway analysis, and Protein-protein interaction network analysis.

**Results:**

Through identification and detailed analysis of the obtained data, we found significant differences in the expression of Hsa-miR-320d, EBV-miR-BART22, and EBV-miR-BART2-3p in blood samples from patients with different EBV-related febrile diseases. We found that the expression levels of Hsa-miR-320d, EBV-miR-BART22, and EBV-miR-BART2-3p in plasma are indicative of determining different disease types of EBV-related febrile diseases, while EBV-miR-BART22 and EBV-miR-BART2-3p may be potential therapeutic targets.

**Conclusion:**

The expression levels of Hsa-miR-320d, EBV-miR-BART22, and EBV-miR-BART2-3p suggest that they may be used as transcriptional features for early differential diagnosis of EBV-related febrile diseases, and EBV-miR-BART22 and EBV-miR-BART2-3p may be potential therapeutic targets.

## Highlights

• The team is the first to collectively study febrile illnesses associated with Epstein-Barr virus (EBV) infection, including EBV-induced fever, infectious mononucleosis (IM) and chronic EBV infection (CAEBV), EBV-induced phagocytic syndrome (EBV-HLH), filling a gap in the field of EBV research.
• The team collected 110 plasma samples, including healthy individuals, a significant number of samples for a relatively uncommon infectious disease.
• The team aimed to discover a pattern of plasma miRNA expression to establish an early diagnosis indicator to determine the certain type of the disease among the EBV-associated febrile diseases.
• The team identified the ebv-miR-BART2-3p, ebv-miR-BART22, and hsa-miR-320d as novel plasma microRNA expression features to be potential biomarkers for the early diagnosis of Epstein-Barr Virus-induced febrile diseases.

## 1 Introduction

### 1.1 Background

Clinically, Epstein-Barr virus (EBV) infection is often overlooked due to its character to present as an asymptomatic course or a self-limiting illness resembling a flu syndrome distinguished by fever, malaise, headache, lymphadenitis, and pharyngitis that usually resolves spontaneously within a few weeks [1,2]. However, some illnesses that are associated with EBV infection, such as infectious mononucleosis (IM), EBV-associated Hemophagocytic Lymphohistiocytosis (EBV-HLH) and Chronic Active Epstein-Barr Virus (CAEBV) infection, are characterized by persistent or intermittent fever and can suddenly worsen or progress to such as lymphoma [3]. Primary EBV infection is generally asymptomatic and commonly occurs during preadolescence; however, it can manifest as infectious mononucleosis (IM) when contracted during puberty or adulthood (30%–50%) [4]. Its symptoms include fever, skin rash, lymphadenopathy, hepatitis, lymphoproliferative disorders, and so on [[5], [6], [7]]. It is also linked to EBV-HLH with worse prognosis, whose mortality rates are up to 40% [8,9]. Chronic Active Epstein-Barr Virus infection (CAEBV), with typical symptoms including lymphadenopathy, fever, anemia and joint pain [10,11], affects people globally, with incidence rates ranging from 0.34 to 3.3 per 100,000 in Asia and a higher prevalence in some regions such as China and Japan [[12], [13], [14]]. This underscores the importance of early diagnosis of EBV to ensure the best possible outcomes for those afflicted with this disease.

EBV is implicated in multisystem diseases and autoimmune diseases, ranging from minor to life-threatening diseases, including a variety of respiratory, cardiovascular, genitourinary, neurological disorders, rheumatoid arthritis, Crohn's disease, and multiple sclerosis [[15], [16], [17], [18]]. Additionally, as one of the earliest known tumorigenic viruses, EBV is associated with lymphoma, nasopharyngeal carcinoma, and gastric cancer, and other neoplasms [[19], [20], [21]].

The Epstein-Barr virus is primarily transmitted through saliva, latently residing in B lymphocytes for life, only to be released into the bloodstream when the host's immune system is compromised [[22], [23], [24]]. Consequently, the activity of the virus is subject to the fine-tuned control of the host's immune system. Due to the persistent tug-of-war between the host and the virus, the potential pathogenicity of EBV has always been of great interest to researchers [[25], [26], [27]]. The chronic viral infections and the mild inflammation on the long-term basis have become the cutting edge of the research on Epidemiology as we walk into a post-covid era [28,29], which leads to this field including EBV infection a research hotspot.

In addition, as the earliest discovered viral miRNA, EBV-encoded microRNA（mirnas), it is of great reference value to study EBVmirnas and their mechanism of action in patients with EBV-related febrile diseases [30] MiRNAs are a class of small RNAs that are 17–24 nt in length and could modulate gene expression by degrading miRNA or inhibiting translation [31]. In recent years, miRNAs have garnered attention for their potential as diagnostic biomarkers for various kinds of infective diseases [32,33]. Furthermore, tissue-specific EBV infections make EBV-related tumors usually express only limited virus-encoded proteins, suggesting that tumorigenesis and progression are likely to be influenced by EBV miRNA and other related factors [34]. Moreover, miRNAs may play a similar part in the diagnosis and treatment of other diseases [35,36]. Despite this, the role of miRNA in the occurrence and development of EBV-associated diseases is still incompletely understood.

In this study, we focused on screening specific plasma miRNA expression features to identify different febrile diseases associated with EBV. Furthermore, we aimed to explore a pattern of plasma miRNA expression to find an early diagnosis indicator to determine the certain type of the disease among the EBV-associated febrile diseases. We proposed a hypothesis and preliminarily verified that among the candidate miRNAs, the EBV-miR-BART2-3p, EBV-miR-BART22, and hsa-miR-320d may not only distinguish EBV infection persons from healthy controls but also separate EBV-HLH, CAEBV, and IM from EBV-associated febrile diseases as well. Thus, we identified novel plasma microRNA expression features to provide potential for the early differential diagnosis of EBV-associated febrile diseases.

## 2 Materials and methods

### 2.1 Study design

**Fig. 1:**
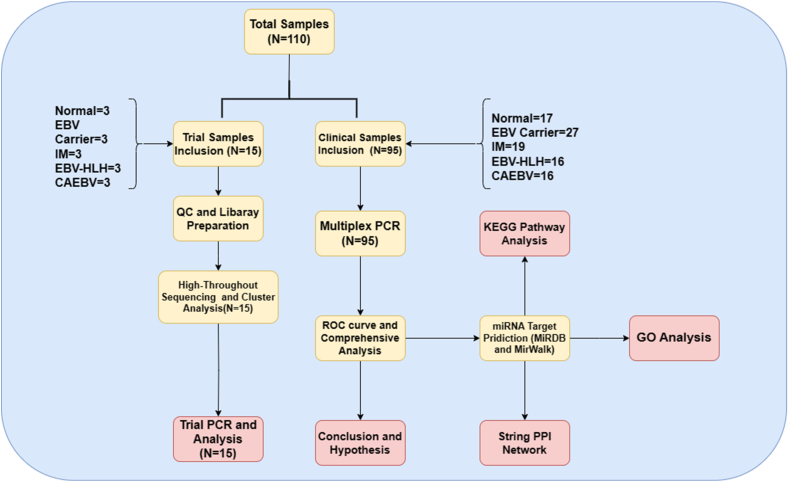
**| The General Process Abstract** This figure shows our study's process. We first got clinical samples that met the blood test index and were diagnosed. miRNA samples and data were obtained after RNA extraction and quality control of their blood samples. We then analyzed High-throughput sequencing and judged by clustering diagram with the trial Samples (N = 15), and then miRNA targets were predicted (N = 10) and selected (N = 3) with the help of trial PCR and analysis. Later the Multiplex PCR were performed using the total samples (N = 95) to further explore the clinical value of the candidate miRNAs along with the bioinformatics analytic protocols (including GO, KEGG analysis and PPI network.).

To identify significant differentially expressed microRNAs from EBV-associated diseases, we employed high-throughput sequencing (HiSeq) and quantitative real-time polymerase chain reaction (qRT-PCR). Out of 110 total enrolled participants, we first collected 15 participants plasma samples to do the high-throughput sequencing. The sequencing was implemented after the Quality Check (QC) and the library preparation. The collected samples could be classified as 5 groups, 3 normal people and 12 EBV-infected people (which were equally distributed into IM, Carrier, CAEBV, and EBV-HLH patients, n = 3). We then conducted subsequent cluster analysis to compare the significantly expressed miRNAs between the groups, as evidenced by the heat map structure. We cross-examined miRNAs expressed differently in all samples and used Venn diagrams to explore potential new targets to be revealed. Real-time quantitative PCR experiments were conducted among the 15 participants to assess the discriminatory value of the 10 differentially expressed miRNAs initially identified through high-throughput sequencing in patients with EBV-associated fever between different diseases and symptoms. The PCR results were analyzed using the 2^−ΔΔCT^ method to examine the expressions of the miRNAs among the different disease species. Subsequently, fold differences were observed and preliminary evidence of validation was obtained. The results of the two validations were compared, and hsa-miR-320d, EBV-miR-BART2-3p, and EBV-miR-BART22 were selected for subsequent multiplex PCR among the total 95 participants to explore their potential as markers for distinguishing different diseases caused by EBV fever. Our comprehensive analysis including the ROC curve revealed these candidate miRNAs were significantly dysregulated in all EBV-Carrier, EBV-HLH, IM, and CAEBV patients. The three miRNAs met our requirements were chosen, which were then used in gene ontology and Kyoto Encyclopedia of Genes and Genomes datasets to build a model - including a protein-protein interaction network - to theorize our findings (Fig. 1).

### 2.2 Sample collection criteria

A total of 110 participants were enrolled in this study, including healthy individuals, EBV carriers, and patients with EBV-HLH, IM, and CAEBV, recruited from Huashan Hospital Affiliated to Fudan University between November 2019 and July 2021. To be included, participants had to meet the following criteria: (1) temperature ≥37.3 °C and EBV-DNA load >1 × 10^3^ copies/10^6^ cells in peripheral blood; (2) age >15-years-old; (3) conformance to the standard for diagnosis of EBV carriers, IM, EBV-HLH and CAEBV, as diagnosed by clinicians based on the latest clinical guidelines [37]. And the related laboratory examinations we've performed with the standards we followed were shown in Table 1. We collected 95 participant samples which could be classified as 5 groups, 17 normal people and 78 EBV-infected people which were separately distributed into IM (N = 19), EBV Carrier (N = 27), CAEBV (N = 16), and EBV-HLH (N = 16) patients building on the diagnostic standards above. The basic information of the participants could be referred to Table 2.

**Table 1:** **Serological Diagnosis Information** This table graphically describes the Serological Diagnosis tests we conducted on the patients, and the standards we followed to diagnose the patients for the related diseases, all of the serological Diagnosis standards match the latest clinical guidelines [37].

Diseases	Serological Diagnosis Standard
EBV-HLH	fibrinogen＜1.5 g/L
	hemoglobin＜90 g/L
	blood platelet＜100 × 10^9/L
	neutrophil＜100 × 10^9/L
	ferritin≥500ug/L
IM	EBVCA IgM Antibody Positive
	EBVCA IgG Antibody Positive
	EBNA IgG Antibody Positive
CAEBV	EBVCA IgM Antibody Positive
	EBVCA IgG Antibody Positive
	EBNA IgG Antibody Positive
	EBV-DNA Positive
EBV Carrier	EBV-DNA Positive

**Table 2:** **Basic patient information** This table graphically describes the basic information we collected on the patients; the age and gender of the healthy people (Normal group) are not taken into account.

Group Name:	Normal	EBV carrier	IM	EBV-HLH	CAEBV
**Sample size**	**17**	**27**	**19**	**16**	**16**
**Gender (Quantity)**	**N/A**	**22(M), 5(F)**	**10(M), 9(F)**	**11(M), 5(F)**	**10(M), 6(F)**
**Age (Quantity)**	**N/A**	**15**–**40(6), 40–60(6), >60(15)**	**15**–**40(17), 40–60(2)**	**15**–**40(6), 40–60(7), >60(3)**	**15**–**40(**6**), 40–60(5), >60(3)**

Among total plasma samples, for the trial, plasma samples from 12 patients and 3 healthy volunteers were analyzed by high-throughput sequencing (Illumina NextSeq 500) and initial qRT-PCR on 10 candidate miRNAs. For further validation, plasma samples from all 78 patients and 17 healthy volunteers were tested by qRT-PCR.

Written informed consent was obtained from all participants, and this study has been approved by the Research and Ethical Committee of Huashan Hospital Affiliated to Fudan University. (No. KY2020-1122)

### 2.3 Plasma preparation and micro RNA extraction

Blood was drawn from the above-mentioned subjects recruited into this study. About 1 ml of peripheral whole blood were collected from each individual into free-enzyme-containing ethylenediaminetetraacetic acid (EDTA) tubes. Following the protocol provided by the producers, the blood was collected into EDTA tubes for centrifugation at 12,000 g for 10 min at 4 °C. Finally, the precipitate was collected into 1.5 ml Eppendorf (EP) tubes and stored at −80 °C for further analysis.

After the preparation of the plasma, the miRNAs were extracted using the TRIZOL method, following the producers’ protocol. We add 0.75 m L of TRIZOL reagent and 0.2 m L of chloroform (Shanghai Chemical Reagent Co., Ltd, China) to the samples and mix well, let it stand for 3–5 min; centrifuge for 10 min at 4 °C and 12,000 g centrifugal force; aspirate the supernatant and transfer it to another EP tube, then add 0.5 m L of isopropanol and mix well. Incubate at −20 °C overnight; centrifuge again at 4 °C and 10,000 g for 10 min, discard the supernatant, then add 1 m L of 75% ethanol, mix well, and continue centrifugation at 4 °C and 10,000 g for 10 min; then air dry at room temperature, add 30 μL of DEPC-treated dd H_2_O water to dissolve the RNA, and store at −80 °C. Storage.

### 2.4 Quality Check (QC) and MiRNA sequencing analysis

The concentration of the plasma RNA from tissue specimens was assessed by measuring the optical density at 260 nm (OD260). And the purity of plasma RNA was evaluated by the ratio of the absorbance at OD260 to OD280. The procedures above were performed by a Nano-Drop 1000 spectrophotometer.

The miRNA sequencing was performed on 15 plasma samples (12 patients with EBV-infection and 3 healthy volunteers) for the trial. The small RNA sequencing library was prepared using the NEB Multiplex Small RNA Library Prep Set for Illumina (NEB. Inc, USA). Approximately, 100 ng of RNA were used to prepare a small RNA library according to the protocol. After 5′ and 3′ adaptor ligation, RNA was reverse-transcribed and amplified using 14 PCR cycles of 98 °C for 10 s and 72 °C for 15 s to generate small RNA libraries. Then, the libraries were validated by loading 1 μl onto the Agilent Technologies 2100 Bioanalyzer to check the size (average molecule length) and purity, and by quantitative PCR using EvaGreen® dye (Jena Bioscience) to verify the concentration. We select PCR amplified fragments among 135 to 155 bp, corresponding to 15–35 nt small RNAs, and Generate single-stranded DNA by denaturation with 0.1 MNaOH, capture on an illumina flow cell, and amplify in situ into clusters. Then, the sequencing was performed on Illumina NextSeq 500 with 51 cycles by Aksomics (Shanghai, China) following the vendor's recommended protocol.

Subsequently, the raw data underwent stringent quality control and this filtering process removed 3′ splice sequences from Clean Reads, leaving tags ≥15 nt in length to form trimmed data which was then compared to the reference genome using bowtie. The miRNA expression threshold was determined by the mean value of Counts per million reads (CMP) in each group exceeding ≥1. MiRNAs considered to be expressed in a group underwent statistical analysis. CPM was calculated as C × 106/N (where C is the number of reads compared to a gene, N is the total number of reads compared to all genes). Differential miRNA expression analysis between groups was performed with edgeR utilizing a threshold value of 1.5-fold difference, P-value ≤0.05, and intra-group CPM mean value ≥ 1 in order to screen for differential miRNAs, and generate visuals such as miRNA clustering plots. Venn diagrams were created to identify miRNAs with more pronounced differential expression.

### 2.5 Quantitative real-time polymerase chain reaction (qRT-PCR) for the validation of miRNAs

In order to validate the candidate miRNAs, two qRT-PCR experiments were performed. The trial qRT-PCR was done including 10 miRNAs (i.e., ebv-miR-BART2-3p, ebv-miR-BART22, ebv-miR-BART2-5p, ebv-miR-BART13-5p, ebv-miR-BART17-5p, ebv-miR-BHRF1-1, hsa-miR-7702, hsa-miR-511-5p, hsa-miR-204-3p, hsa-miR-320d) among the 15 samples, distributed into Normal (N = 3), IM (N = 3), EBV Carrier (N = 3), CAEBV (N = 3), and EBV-HLH (N = 3). The multiplex PCR were employed using the total samples (N = 95), distributed into Normal (N = 17), IM (N = 19), EBV Carrier (N = 27), CAEBV (N = 16), and EBV-HLH (N = 16) Following the extraction of the RNA, a complementary DNA was subsequently synthesized using the TaqMan microRNA Reverse Transcription Kit (USA) in a reverse transcription reaction. To execute qRT-PCR, the miScript SYBR Green PCR kit (Qiagen) was employed, with an ABI7500 instrument (Applied Biosystems) being utilized to carry it out. The PCR was conducted according to the specifically prescribed procedure, which included: 110 °C, 10 min; 40 PCR cycles (110 °C, 10 s; 60 °C, 60 s (collection of fluorescence)); and a melting curve of the PCR products, attained after the amplification reaction was completed through the following process: 110 °C, 10 s; 60 °C, 60 s; 110 °C, 15 s; and slow heating from 60 °C to 99 °C (which was performed automatically by the instrument - Ramp Rate of 0.075 °C/s). Hsa-miR-93-5p was implemented as an internal control to normalize the quantification of RNA. All of the primer sequences used in the qRT-PCR process are shown in Table 3. Finally, the data was statistically analyzed using the 2^–ΔΔCt^ method.

**Table 3:** **Primer Sequences in the qRT-PCR** This table depicts the primer sequences for qRT-PCR. (qRT-PCR: quantitative reverse transcription-polymerase chain reaction; GSP: Gene-specific primer; R: reverse primer.)

miRNA Name		Sequence					
hsa-miR-320d		>hsa-miR-320d: AAAAGCUGGGUUGAGAGGA					
		GSP:5′AAAAGCTGGGTTGAGAGGA3′					
		R:5′CAGTGCGTGTCGTGGAGT3′					
ebv-miR-BART22		>ebv-miR-BART22: UUACAAAGUCAUGGUCUAGUAGU					
		GSP:5′GGGGGTTACAAAGTCATGGTCT3′					
		R:5′GTGCGTGTCGTGGAGTCG3′					
ebv-miR-BART2-3p		>ebv-miR-BART2-3p: AAGGAGCGAUUUGGAGAAAAUAAA					
		GSP:5′GGAAGGAGCGATTTGGAGAA3′					
		R:5′GTGCGTGTCGTGGAGTCG3′					
hsa-miR-93-5p		>hsa-miR-93-5p: CAAAGUGCUGUUCGUGCAGGUAG					
		GSP:5′AACAAGCAAAGTGCTGTTCGT3′					
		R:5′GTCGTATCCAGTGCAGGGT3′					

### 2.6 Bioinformatics analysis

We used different bioinformatics methods to further develop our findings. To predict the genes targeted by differentially expressed miRNAs, MiRHybrid, miRDB (https://mirdb.org/) and miRWalk 2 (http://mirwalk.umm.uni-heidelberg.de/)were used to identify miRNA-binding sites. Finally, the data predicted by both the algorithms were combined and the overlaps were calculated.

The Gene Ontology (GO) terms and the Kyoto Encyclopedia of Genes and Genomes (KEGG) pathway of these differentially expressed miRNA targets were annotated based on FunRich (http://funrich.org/index.html), a stand-alone software tool utilized primarily for functional enrichment and interaction network analysis of genes and proteins. Protein-protein interaction (PPI) network was drafted as well on STRING, The Search Tool for the Retrieval of Interacting Genes (STRING; http://string.embl.de/) which is a biological database designed to construct a PPI network. Furthermore, the results of the analysis are represented graphically in the form of Venn, Bar, Column, Pie and Doughnut charts in this research.

### 2.7 Statistical analysis

Data were analyzed using either a one-way or a two-way analysis of variance (ANOVA) with post hoc Tukey's test. P < 0.05 was deemed as statistically significant. Data are presented as mean ± standard deviation (SD), and graphed using Prism 9 (Inc., La Jolla, CA, USA).

## 3 Results

### 3.1 High-throughput sequencing results of 15 participants identified 10 candidate MiRNAs

**Fig. 2:**
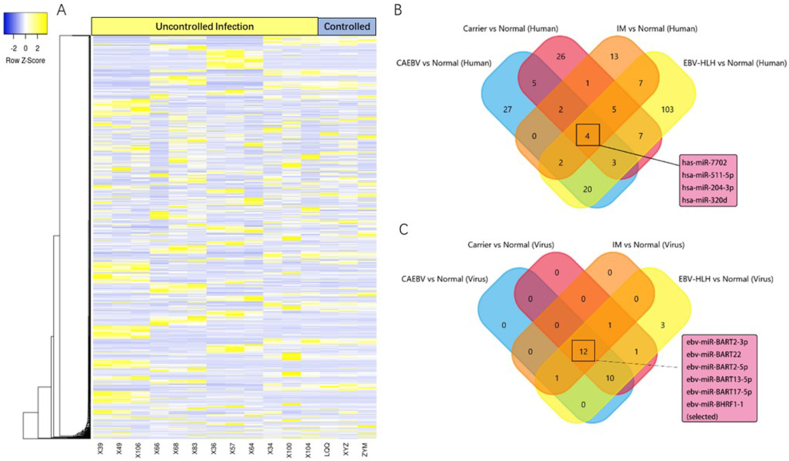
**The screening process of 10 candidate miRNA** Heatmap was created based on differently expressed miRNAs, and classified unorderly based on the cluster algorithm. In picture A, yellow and blue colors represent upregulated and downregulated expression of genes, respectively. Ten candidate miRNAs obtained by cross-referencing key targets using high-throughput gene sequencing showed significant differences between Normal, Carrier, IM, CAEBV, and EBV-HLH groups. In picture B, we conduct the human miRNA's screening. The four groups were CAEBV and Normal (Human), Carrier and Normal (Human), IM and Normal (Human), EBV-HLH and Normal (Human). Each of these groups compared the coincident parts, and the four groups compared the specifically combined parts again, and finally only four miRNAs remained. In picture C, we conduct the virus miRNA's screening. The four groups were CAEBV and Normal (Virus), Carrier and Normal (Virus), IM and Normal (Virus), EBV-HLH and Normal (Virus). Each of these groups compared the coincident parts, and the four groups compared the specifically combined parts again, and twelve miRNAs remained. We went through evidence-based screening and discussion, keeping six target miRNAs for further Polymerase Chain Reaction (PCR) proof. The selected miRNAs were discovered to be relative in the chronic, long-term infection of EBV and the inflammation-cancer transform system [38,39].[38,39]. (For interpretation of the references to color in this figure legend, the reader is referred to the Web version of this article.)

We collected 15 participants samples which could be classified as 5 groups, 3 normal people named as “controlled infection group” and 12 EBV-infected people (which were equally distributed into IM, Carrier, CAEBV, and EBV-HLH patients, n = 3) named as “uncontrolled infection group”. We then conducted subsequent cluster analysis to compare the significantly expressed miRNAs between the groups, as evidenced by the heat map structure depicted in Fig. 2A. We then cross-examined miRNAs expressed differently in all samples and used Venn diagrams to explore potential new targets to be revealed. As illustrated in Fig. 2 B and C, 10 miRNAs (i.e., ebv-miR-BART2-3p, ebv-miR-BART22, ebv-miR-BART2-5p, ebv-miR-BART13-5p, ebv-miR-BART17-5p, ebv-miR-BHRF1-1, has-miR-7702, hsa-miR-511-5p, hsa-miR-204-3p, hsa-miR-320d) were verified to be differentially expressed in different types of EBV-infection upon high-throughput sequencing of miRNA via an Illumina sequencer. This analysis yielded significant differences in miRNA transcription levels among the various types of EBV infection and thus merits further research and data processing.

### 3.2 PCR in trial suggested hsa-miR-320d, EBV-miR-BART22, and EBV-miR-BART2-5p as potential biomarkers for the identification of the EBV-associated disease

Real-time quantitative PCR experiments were conducted to assess the discriminatory value of the 10 differentially expressed miRNAs initially identified through high-throughput sequencing in patients with EBV-associated fever between different diseases and symptoms. The PCR results were analyzed using the 2^−ΔΔCT^ method to examine the expressions of the miRNAs among the different disease species. Subsequently, fold differences were observed and preliminary evidence of validation was obtained. The results of the two validations were compared, and hsa-miR-320d, EBV-miR-BART2-3p, and EBV-miR-BART22 were selected for subsequent multiplex PCR to explore their potential as markers for distinguishing different diseases caused by EBV fever.

### 3.3 Correlation of the expression of hsa-miR-320d, EBV-miR-BART2-3p, and EBV-miR-BART22 with various syndromes of patients with EBV infection causing fever

**Fig. 3:**
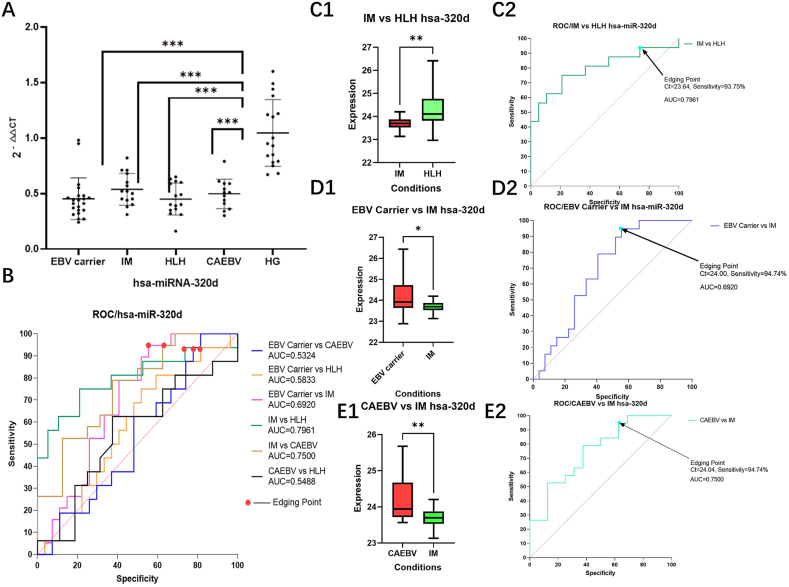
**Comparison of hsa-miR-320d relative gene expression 2**^**-△△CT**^**and ROC curve analysis results between different disease groups and healthy human groups** Analysis of hsa-miR-320d expression revealed distinct diagnostic significance among various EBV-associated febrile disease groups. Strikingly, a ct critical value of 23.64 in the IM and EBV-HLH groups demonstrated a diagnostic sensitivity of 93.75%, along with an AUC of 0.7961, suggesting that EBV-infected febrile patients with ct values > 23.64 may be more likely identified as EBV-HLH patients. Furthermore, a critical value of ct at 24.00 in the EBV Carrier and IM groups yielded 94.74% diagnostic sensitivity and an AUC of 0.6920, implying that EBV-infected febrile patients with ct values < 24.00 may be more likely identified as IM patients. In addition, an AUC of 0.7500 was discovered when the ct critical value in the CAEBV and IM groups was 24.04, suggesting that when ct values are <24.04, EBV-infected febrile patients may be more likely identified as IM patients. Taken together, these results implicate hsa-miR-320d as a promising marker for the initial differential diagnosis of various EBV- associated febrile diseases.. (The label of HLH in the figure serves as the EBV-HLH group.)

As depicted in Fig. 3, analysis of Hsa-miR-320d miRNA qRT-PCR revealed significant differences in miRNA expression between the Normal group, EBV Carrier group, IM group, EBV-HLH group, and CAEBV group (P < 0.001), and the exact results were illustrated in Fig. 3A, C1, D1, E1. Moreover, AUC for this gene in the IM group surpassed that in the EBV-HLH and EBV Carrier groups, as well as the CAEBV group, by values greater than 0.65, illustrating the potential of hsa-miR-320d as a tool for diagnosis of EBV-associated fever-like diseases (Shown in Fig. 3B, C2, D2, E2).

**Fig. 4:**
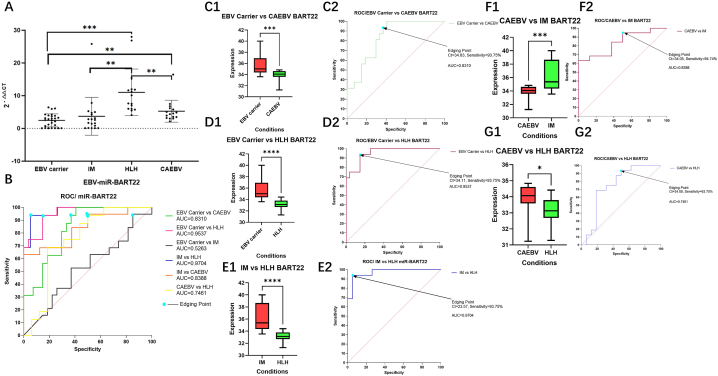
**Comparison of 2**^**-△△CT**^**of EBV-miR-BART22 relative gene expression between different disease groups and results of ROC curve analysis** Analysis of ebv-miR-BART22 expression in EBV-associated febrile diseases and its discriminatory role in differential diagnosis was evaluated via qRT-PCR assay. Multivariate comparison of miR-BART22 between all EBV febrile diseases (EBV Carrier group, IM group, EBV-HLH group and CAEBV group) showed significant differences between EBV Carrier group and EBV-HLH group; CAEBV group and IM group; CAEBV group and EBV-HLH group (P < 0.001 or P < 0.01). In addition, comparison between the four groups revealed that the AUC of miR-BART22 expression was higher than 0.7 (AUC>0.7), indicating it as a moderately reliable model for initial diagnostic identification in EBV-associated febrile diseases. Analysis of ebv-miR-BART22 cut-off ct values provided a diagnostic sensitivity of 93.75%–94.74%, with an AUC between 0.7451 and 0.9704. Thus, our findings suggest that miR-BART22 PCR results may be a potential biomarker for the differential diagnosis of EBV-related febrile diseases.. (The label of HLH in the figure serves as the EBV-HLH group.)

Further analysis of PCR results shown in Fig. 4 indicated that a threshold value of ct values < 24 might be used as a differential diagnosis of IM. Analysis of the expression of EBV-miR-BART22 by MiRNA qRT-PCR revealed highly significant differences between the EBV carrier group and EBV-HLH and CAEBV groups (P < 0.0001; P < 0.001), and significant differences between the IM and EBV-HLH groups (P < 0.001). Moreover, the EBV-HLH and CAEBV groups also displayed significant differences (P < 0.001). These results suggest that EBV-miR-BART22 may be useful in distinguishing different diseases caused by EBV fever, and the exact results were illustrated in Fig. 4A, C1, D1, E1. ROC curve analysis revealed high comparative AUC values between the EBV carrier group and the CAEBV, EBV-HLH and IM groups (AUC>0.7), indicating the potential of miR-BART22 as a diagnostic marker. Further analysis of the ct (cycle times) value of EBV-miR-BART22 revealed that the threshold value as a differential diagnosis of EBV-HLH may be less than 34.05 (Shown in Fig. 4B, C2, D2, E2).

**Fig. 5:**
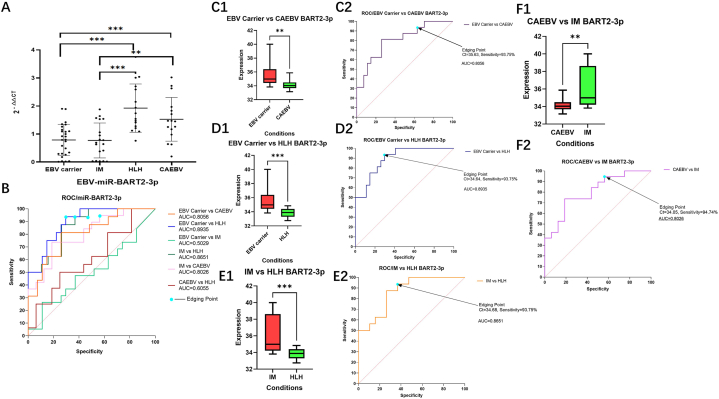
**Comparison of 2**^**-△△CT**^**of EBV-miR-BART2-3p relative gene expression between different disease groups and results of ROC curve analysis** The Analysis of ebv-miR-BART2-3p expression in EBV-associated febrile diseases was conducted by PCR assay and miRNA qRT-PCR assays in 78 symptomatic patients and 17 healthy comparison subjects. Significant differences in gene expression were found between the four disease groups (EBV Carrier, CAEBV, IM and EBV-HLH) (P < 0.001 or P < 0.01). Using a multivariate model, the predictive potential of ebv-miR-BART2-3p was further analyzed and indicated a moderately reliable model (AUC >0.7). Furthermore, critical ct values were used to distinguish the disease groups, including 93.75% diagnostic sensitivity for EBV Carrier versus CAEBV (ct < 35.63), EBV Carrier versus EBV-HLH (ct < 34.64) and IM versus EBV-HLH (ct < 34.68). Additionally, the CAEBV versus IM group showed a 94.74% diagnostic sensitivity with an AUC of 0.8026 at a ct value of <34.05. These results support the notion that ebv-miR-BART2-3p expression may be a potential useful biomarker in differentiating between various EBV-associated febrile diseases as indicators for differentiation of different diseases caused by EBV fever.. (The label of HLH in the figure serves as the EBV-HLH group.)

Analysis of relative gene expression of EBV-miR-BART2-3p by MiRNA qRT-PCR graphed in Fig. 5 revealed markedly divergent results between the EBV carrier group and EBV-HLH and CAEBV groups (P < 0.0001; P < 0.001). Furthermore, a substantial discrepancy was also detected between the IM group and the EBV-HLH and CAEBV groups (P < 0.001; P < 0.01) and the exact results were illustrated in Fig. 5A, C1, D1, E1. ROC curve analysis yielded a high AUC of EBV-miR-BART2-3p expression between the respective disease groups (AUC >0.7), implying the potential of miR-BART2-3p in accurately diagnosing illnesses associated with EBV infection, Additionally, the PCR results for EBV-miR-BART2-3p revealed that a ct value < 35.63 was more likely to be attained in the CAEBV or EBV-HLH groups (Shown in Fig. 5B, C2, D2, E2). Collectively, these findings suggest that EBV-miR-BART2-3p might serve as a dependable diagnostic marker for distinguishing EBV-related febrile diseases.

### 3.4 Gene and miRNA enrichment analysis

**Fig. 6:**
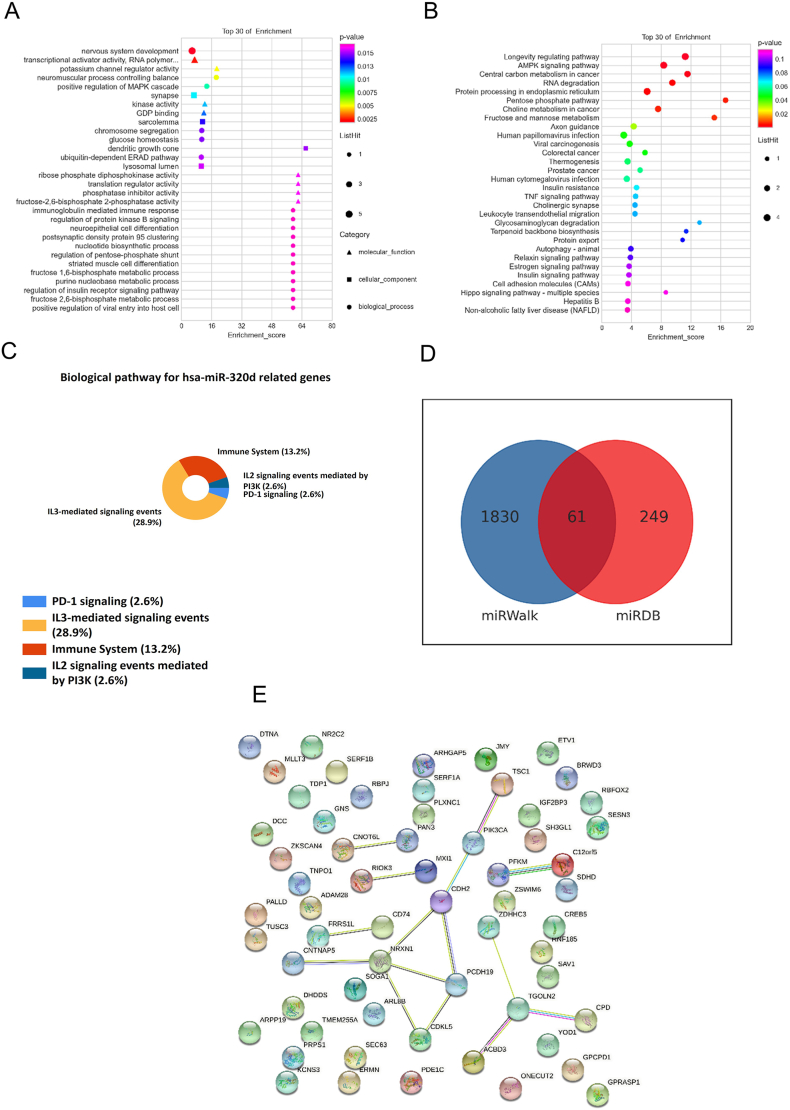
**Enrichment Analysis Status of hsa-miR-320d-related Genes**Fig. 6 shows the enrichment analysis status of hsa-miR-320d-related genes. hsa-miR-320d-associated genes are analyzed from three perspectives: MF, CC, and BP in A. Both B and C are analyzed from the perspective of biological pathways. D shows the screening of miRNA-related genes. E further reveals the hsa-miR-320d-related genes' interaction in biological processes through the PPI network.

**Fig. 7:**
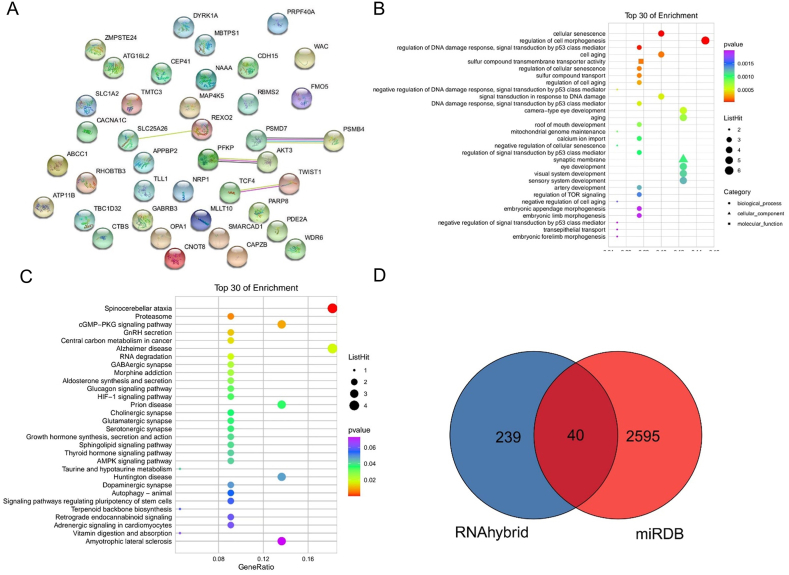
**Enrichment Analysis Status of EBV-miR-BART22 and EBV-miR-BART2-3p-related genes**Fig. 7 shows the enrichment analysis status of EBV-miR-BART22 and EBV-miR-BART2-3p-related genes. A reveals the hsa-miR-320d-related genes' interaction in biological processes through the PPI network pathway. The selected genes are analyzed from three perspectives: MF, CC, and BP in B. In C the genes are analyzed from the perspective of biological pathways. D shows the screening of genes.

Drawing on our findings, we proceeded to investigate the expression and mutations associated with studied miRNAs: hsa-miR-320d, EBV-miR-BART22, and EBV-miR-BART2-3p. To do this, we utilized two online databases, miRWalk and miRDB, to screen a total of genes related to the hsa-miR-320d and, subsequently, employed GO enrichment analysis, including four components of Biological Process (BP), Cell Components (CC), Biological Pathway, and Molecular Function (MF), KEGG enrichment analysis, and PPI network analysis (Fig. 6). In order to elucidate the expression and mutations for EBV-miR-BART22 and EBV-miR-BART2-3p, we implemented the same analyses using miRHybrid and miRDB (Fig. 7)

Drawing on our findings, we proceeded to investigate the expression and mutations associated with studied miRNAs: hsa-miR-320d, EBV-miR-BART22, and EBV-miR-BART2-3p. To do this, we utilized two online databases, miRWalk and miRDB (Fig. 6D), to screen a total of genes related to the hsa-miR-320d and, subsequently, employed GO enrichment analysis, including four components of Biological Process (BP), Cell Components (CC), Biological Pathway, and Molecular Function (MF), the results were performed in Fig. 6A. KEGG enrichment analysis were conducted to discover the miRNA related genes in forms of the pathway networks, and the results were shown in Fig. 6B and C. And PPI network analysis was performed to evaluate the expressions of key proteins, the results were shown in Fig. 6E. In order to elucidate the expression and mutations for EBV-miR-BART22 and EBV-miR-BART2-3p, we implemented the same analyses using miRHybrid and miRDB (Fig. 7D). The PPI network results were shown in Fig. 7A. The analysis of GO enrichment in BP, MF and CC were depicted in Fig. 7B, the KEGG pathway results were depicted in Fig. 7C.

To characterize the biological functions of the genes related to the candidate miRNAs between the patients with EBV infections and healthy people. GO analysis was performed. The top enriched GO terms in BP comprised processes predominately related to the immune response (Fig. 6, Fig. 7B). Most candidate miRNA related genes involved in the immune response were downregulated in patients with EBV infections. In Fig. 6B, it is shown that hsa-miR-320d-related genes have a pronounced association with pathways connected to cancer and inflammation, such as AMPK, MAPK, and PD-1. Furthermore, the effects of EBV-miR-BART22 and EBV-miR-BART2-3p related genes on cancer and inflammation related pathways, such as p53, are highlighted in Fig. 7C. Interestingly, genes related to inflammation-cancer system were vastly enriched in the samples of EBV-infected patients. Several most enriched GO terms in CC and MF were also identified (Fig. 6, Fig. 7B). Additionally, KEGG pathway analysis revealed that most genes related with candidate miRNAs in both healthy and patients with EBV infections samples were associated with various pathways, including the pathway for inflammation and tumorigenesis (Fig. 6, Fig. 7C), including PD-1 signaling pathway, HIF-1 signaling pathway, AMPK signaling pathway, etc. To assess the protein expression profiles associated with EBV-infected patients, PPI network analysis was performed. As it showed in Fig. 6, Fig. 7A, the proteins related in the inflammation-cancer, such as CD74, PIK3CA, AKT3 have the potential to perform in a network to act in the transformation of the chronic inflammation into tumorigenesis.

## 4 Discussion

Circulating miRNAs are widely distributed throughout the body, interacting with tissues and organs [40]. Bioinformatics studies have demonstrated that the target gene hsa-miR-320d is involved in the pathogenesis of inflammatory bowel disease [41], with elevated concentrations observed in the blood under inflammation-prone conditions [42]. Furthermore, this miRNA may be specific to systemic inflammatory disorders, including knee osteoarthritis [43]. It is important to consider the possibility that this miRNA may be involved in systemic diseases rather than solely musculoskeletal conditions. Previous reports have shown that hsa-miR-320d is deeply implicated in the MAPK signaling pathway and is associated with apoptosis and inflammation [44]. This pathway is also implicated in the development of various malignancies [45]. Thus, modulated miRNA expression in the plasma could influence the progression of EBV infection through the MAPK signaling pathway, facilitating the transition from EBV-associated fever to disease. This suggests that plasma expression of hsa-miR-320d may interfere with the development of inflammation accompanying EBV infection by regulating inflammatory pathways such as MAPK in EBV-infected cells, and that this miRNA has a phenotypic signature in the direction of inflammation regulation, thereby indicating the lack expression of this miRNA suggests the absence of host defense against EBV in these individuals.

A research team studying clear cell renal cell carcinoma (ccRCC) recently built a miRNA-mRNA dysregulation network, which revealed that hsa-miR-320d had the largest degree and a high level of anticorrelated mRNAs, implying its critical role in the development of ccRCC [46]. The dysregulated mRNAs of hsa-miR-320d are related to cell localization and function in electron transporters and catalytic activities. This is in close agreement with our previous study that the expression of this miRNA in EBV-infected cells correlates with the MAPK signaling regulatory pathway of the inflammatory-cancer pathway. Furthermore, this miRNA's involvement in epithelial cell signaling pathways, Helicobacter pylori infection, oxidative phosphorylation, arginine and proline metabolism, and the degradation of valine, leucine, and isoleucine was also observed. Noteworthy, the enrichment of hsa-miR-320d was identified in the kidneys, lungs, liver, and brain tissues [46]. Additionally, previous studies have shown that hsa-miR-320d has inhibitory effects on the proliferation of diffuse large B-cell lymphoma cells, suggesting its potential contribution to the immune system's anti-virus response. These findings suggest that hsa-miR-320d may affect EBV infection and inflammation, potentially explaining its expression in plasma as a diagnostic marker for EBV-related febrile diseases [47]. Combing these with our results, this not only guide our further investigation into the connection between hsa-miR-320d and EBV-related febrile diseases, but also shed light on the differential expression of hsa-miR-320d in such diseases, thus validating its ability to serve as a diagnostic marker for EBV-related febrile disorders.

Previous studies have focused on the expression and associated phenotypes of EBV-miR-BART22 in gastric cancer patients and liver transplant recipients [48,49]. One prominent study revealed that EBV-miR-BART22 was highly expressed in EBV-associated gastric carcinoma (EBVaGC) cell lines. Furthermore, it was observed that higher levels of EBV-miR-BART22 expression in primary EBVaGC specimens correlated with a higher likelihood of lymph node metastasis and poorer overall 5-year survival. It was hypothesized that EBV-miR-BART22 could promote cell migration and invasion by down-regulating E-cadherin and up-regulating the Wnt signaling pathway via targeting Dickkopf 1 (DKK1) [50]. Subsequently, a recent paper highlighted an additional EBV-miR-BART22-induced mechanism behind nasopharyngeal carcinoma (NPC) cisplatin chemoresistance (DDP): stimulation of tumor stemness and epithelial-to-mesenchymal transition (EMT) signaling pathway. Moreover, a positive relationship between EBV-miR-BART22 and miR-4721 was also established, proposing that EBV-miR-BART22 may serve as a dominator upstream of miR-4421 and thereby influence NPC progression [51]. And another research echoes with this suggestion, showing that the EBV-miR-BART22 may push the process of EMT signaling pathway through the combinations of genes [52]. The current researches indicate that the EBV-miR-BART22 may target at the related genes or miRNAs to accelerate the process of the epithelial-to-mesenchymal transition signaling pathway. In an inflammation-tumor system, the combination of the miRNA and genes performs as a balancing system to influence the process of tumorigenesis. So, the EBV-miR-BART22 may function as a bridging factor combining the infections and cancers, pushing the chronic inflammations in to tumors. In inflammatory-cancer-related diseases like EBVaGC and NPC, EBV-miR-BART22 exhibits a role for human miRNA expression, thus it is reasonable to assume that in EBV-associated febrile diseases, EBV-miR-BART22, a miRNA of viral origin, may interfere with hsa-miR-320d, a miRNA of human origin previously studied by us, and thus interfere with the inflammatory process of EBV-infected cells and affect the type of EBV-associated febrile diseases. Taken together, these findings suggest that EBV-miR-BART22 could be implicated in aberrant expression of immune cell genes during EBV infection, potentially via mediating the misexpression of normal human miRNA. Under clinical situations, we can imply our findings in the prediction of the EBV-HLH, IM, and CAEBV diseases. In a clinical setting, these findings could be utilized to predict the development of EBV-HLH, IM, and CAEBV diseases. Plasma miRNA examination could serve as a potential implementation of these findings in clinical practice. Additionally, based on previous research, EBV-miR-BART22 may have the ability to influence the expression of key genes involved in the EMT process. Therefore, it is plausible to suggest that EBV-miR-BART22 may also impact inflammation during viral infection by influencing related genes and miRNAs.

Recent investigations have demonstrated that EBV-miR-BART2-3p targets Unc-51-like kinase 1 (ULK1), resulting in a decline in protein markers associated with epithelial-mesenchymal transformation (EMT) as well as a diminishment of EMT and migration [53]. In this regard, EBV-miR-BART2-3p may be participating in the inhibition of autophagy. Recent research suggests that the combination of the EBV-BART2-3p with the ULK1 may block the revitalization of the ULK1 from the influence of the AMPK protein [54]. Changes in the expression of this miRNA could potentially lead to excessive proliferation of immune cells, disrupting the immune system and worsening EBV infections, which may increase the risk of febrile diseases caused by EBV. Our findings in this study support this hypothesis. Similar to the discovery on the EBV-miR-BART22, the EBV-miR-BART2-3p shows its unneglectable functions involving the EMT pathway. This may serve far more than a coincidence but indicates a possibility that the combination of miRNA and certain genes such as ULK1 and DDK1 may become an influencing, balancing factor controlling the transformation from chronic inflammation to chronic tumorigenesis. In clinical practice, the expression of the EBV-miR-BART2-3p may serve as an indicator for physicians to foresee the risk of the EBV infection turning into much complex infective diseases such as EBV-HLH, IM, and CAEBV.

We screened three miRNAs, hsa-miR-320d, EBV-miR-BART22, and EBV-miR-BART2-3p, which have diagnostic value for differentiating EBV infection-associated fever between different diseases and different symptoms. The results suggest that hsa-miR-320d, EBV-miR-BART22, and EBV-miR-BART2-3p may have potential diagnostic values for differentiating EBV infection-associated fever syndromes. Analysis of three miRNAs, hsa-miR-320d, EBV-miR-BART22 and EBV-miR-BART2-3p in EBV infection-associated fever revealed a significant differential expression in gene expression between EBV infection-associated fever diseases, symptoms and healthy individuals. Specifically, hsa-miR-320d was observed to have a discriminatory value with a critical ct value of <24, while EBV-miR-BART22 had a critical ct value of <34.05 and EBV-miR-BART2-3p had a critical ct value of <35.63. Our results provide evidence for the potential of these miRNAs as diagnostic tools for differentiating EBV infection-associated fever between different diseases and symptoms. The expression levels and patterns of these miRNAs in plasma can serve as the indicators and the identifiers of the various diseases induced by the EBV infection. The relationship between these three miRNAs and the clinical manifestations of EBV-associated febrile diseases deserves to be explored in greater depth at the mechanistic level. Based on what's illustrated above, we can foresee these miRNAs performs potential in the realm of virus-related cancer as well. The influencing functions of the EBV-miR-BART22 and EBV-miR-BART2-3p in the EMT signaling pathway involving the NPC is a promising research direction.

The findings suggest that the dysregulation of inflammation-cancer system genes in EBV-infected patients is influenced by the differential expression of related miRNAs. Through analysis of the Gene Ontology (GO) and Kyoto Encyclopedia of Genes and Genomes (KEGG) databases, we discovered that hsa-miR-320d interacts with CD274 in the PD-1 signaling pathway, thereby impacting immune regulation and triggering inflammatory responses. Recent studies show that miRNAs encoded by Epstein-Barr virus (EBV) are essential to the febrile disease caused by EBV infection [55]. EBV-miR-BART22 and EBV-miR-BART2-3p may specifically inhibit human miRNA hsa-miR-320d, thus altering CD274 expression and blocking the PD-1 pathway, resulting in excessive activation of T cells and an abnormal inflammatory response that is characterized by increased levels of immune substances, such as IL-10. The variation in EBV miRNA expression leads to different levels of inhibition of hsa-miR-320d and thus to different degrees of T-cell activation and inflammatory response. Such variation determines the severity of EBV febrile disease, which can be classified into different stages, such as EBV carrier, IM, CAEBV, and EBV-HLH based on the degree of EBV miRNA expression and EBV infection. Notably, the degree of T-cell activation and the phenotypic characteristics associated with the highest stage of EBV infection are taken as critical diagnostic points, with EBV miRNA expression providing an important diagnostic reference indicator. T-cell depletion has been used as an important differential diagnostic feature in hemophagocytic syndromes. [56] Furthermore, the two EBV miRNAs may serve as potential targets for treating EBV infection. In summary, our hypothesis suggests that the expression of three candidate miRNAs in EBV-infected cells may play a role in regulating inflammation-related pathways, including the MAPK signaling pathway, TNF signaling pathway, NF-KB signaling pathway, and PD-1/PD-L1 signaling pathway. These miRNAs may influence the symptoms and disease manifestations observed in patients following EBV infection. Additionally, as the miRNA enrichment analysis focuses on the inflammatory-cancer pathway, it is plausible that the transformation from inflammation to cancer in EBV infection could also be regulated by the expression of these three miRNAs. However, further validation and exploration through cellular experiments are necessary to confirm this hypothesis.

Clinical evidence demonstrates that inflammation is integral to EBV-associated febrile disease, and studies suggest that hsa-miR-320d plays a role in controlling the inflammatory response by interacting with CD274 and modulating the PD-1 pathway. By delving further into the mechanism, we postulated that EBV-miR-BART22 and EBV-miR-BART2-3p hinder the expression of hsa-miR-320d, hence leading to increased inflammation and the manifestation of EBV-associated febrile disease. Therefore, hsa-miR-320d expression might be conceived to serve as a predictive marker for EBV-associated febrile diseases in medical practice. However, the contribution of viral miRNAs should also be taken into account and used as an auxiliary indicator. Because the CT value of miRNA hsa-miR-320d alone could be difficult to differentiate EBV-related fever diseases. In this prospect, the relevant miRNAs we studied may interfere with the progression of intracellular inflammation in EBV-infected cells in the form of miRNA-mRNA-cytokine network pathways, thus affecting the phenotype of EBV-associated febrile diseases. Also, since most of the pathways involved are inflammatory-cancer pathways, it is possible that EBV-infected patients could be converted from non-cancerous to cancerous disorders, which is also consistent with clinical fact. These findings not only provide new insights into the expression of EBV miRNAs in EBV-related febrile diseases but also support their utility as pathological indicators for such conditions.

## 5 Conclusion

In conclusion, the expression differences of EBV-miR-BART22, EBV-miR-BART2-3p, and hsa-miR-320d between the EBV carrier group and the EBV-HLH group, as well as between the EBV carrier group and the CAEBV group, are highly significant. This suggests that they may have important implications in distinguishing EBV carriers, infectious mononucleosis (IM), EBV-HLH, or CAEBV. Therefore, their plasma expression levels can serve as diagnostic markers to determine the type of EBV-related fever diseases, with hsa-miR-320d being particularly important. Additionally, EBV-miR-BART22 and EBV-miR-BART2-3p may have potential as therapeutic targets for EBV-related diseases.

## Funding

This research is funded on 10.13039/501100001809National Natural Science Foundation of China (Grant No. 81904072), and The Fifth National Clinical Excellence in Chinese Medicine Funding Project. (Project Number: A2-X2300704)

## Data availability statement

The data associated with this study hasn't been deposited into a publicly available repositor and will be made available on request.

## CRediT authorship contribution statement

**YiFei Xu:** Writing – review & editing, Writing – original draft, Software, Methodology, Investigation, Formal analysis, Data curation, Conceptualization. **Ying Chen:** Software, Formal analysis, Data curation, Conceptualization. **Qingluan Yang:** Software, Resources, Formal analysis, Data curation. **Yuxiang Lu:** Data curation, Conceptualization. **Rui Zhou:** Writing – original draft, Visualization. **Haohua Liu:** Software, Data curation. **Yanjie Tu:** Supervision, Funding acquisition, Formal analysis, Conceptualization. **Lingyun Shao:** Supervision, Resources.

## Declaration of competing interest

The authors declare that they have no known competing financial interests or personal relationships that could have appeared to influence the work reported in this paper.
