# Supplementary material for: Novel plasma microRNA expression features in diagnostic use for Epstein-Barr virus-associated febrile diseases
Source: Heliyon. 2024 Feb 23;10(5):e26810. doi: 10.1016/j.heliyon.2024.e26810 (PMC10912469; doi:10.1016/j.heliyon.2024.e26810)
Supplement: Multimedia component 1 [file mmc1.docx]

Supplementary Material——Supplementary Figure 1


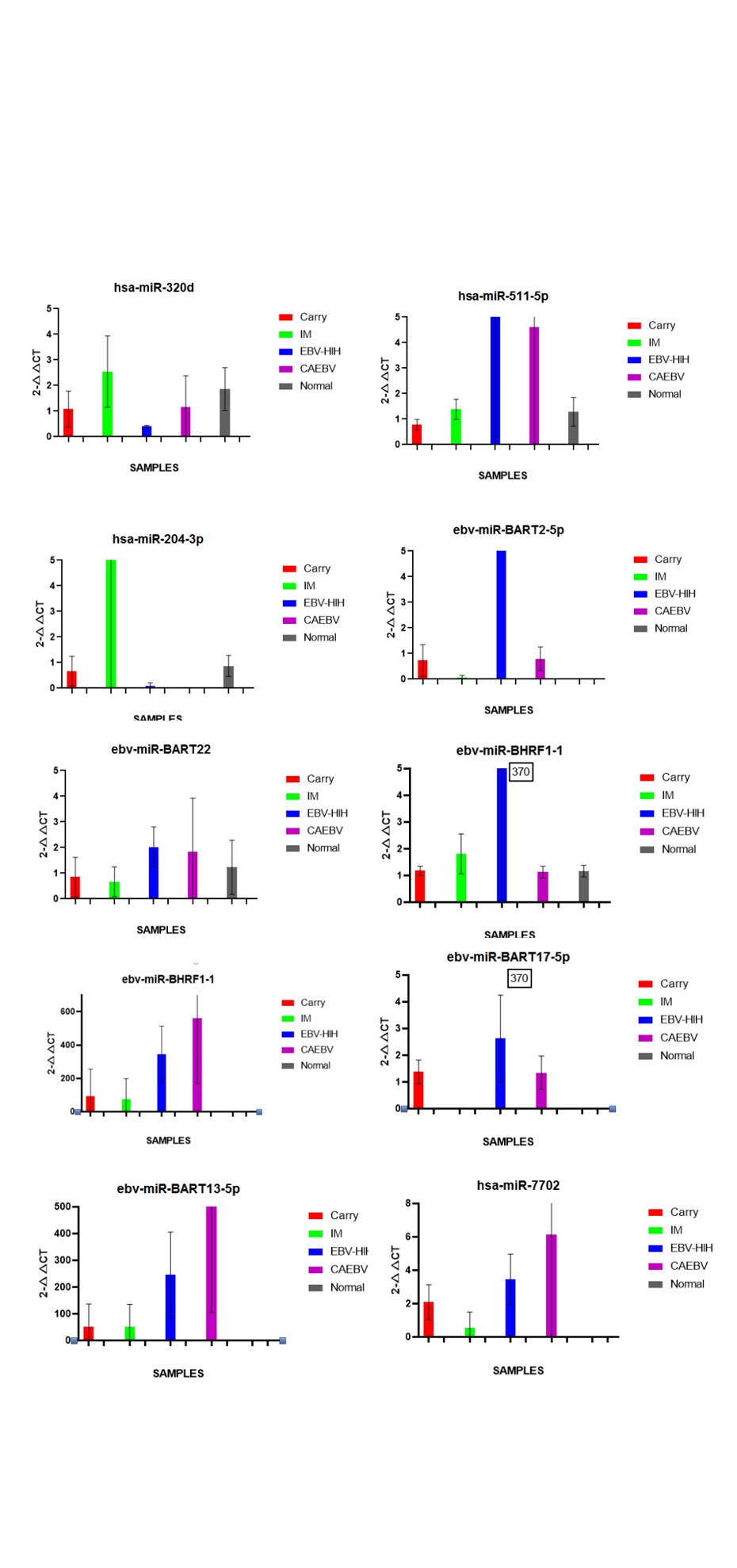


**Supplementary Figure 1 |** **Comparison of candidate miRNAs expression by 2^-△△CT^** **among Epstein-Barr Virus-Associated Febrile Diseases patients and normal people.** The figure depicts the varied miRNAs expressions in the research subjects. Build on the results and the comprehensive analysis, 3 candidate miRNAs were selected: has-miR-320d, ebv-miR-BART22, and ebv-miR-BART2-3p.
